# Prognostic value of pro-adrenomedullin and copeptin in acute infective endocarditis

**DOI:** 10.1186/s12879-020-05655-7

**Published:** 2021-01-07

**Authors:** Rosa Zampino, Domenico Iossa, Maria Paola Ursi, Lorenzo Bertolino, Roberto Andini, Rosa Molaro, Oriana Fabrazzo, Silvia Leonardi, Luigi Atripaldi, Emanuele Durante-Mangoni

**Affiliations:** 1 Department of Advanced Medical and Surgical Sciences, University of Campania “Luigi Vanvitelli”, Naples, Italy; 2 Unit of Infectious & Transplant Medicine, A.O.R.N. Ospedali dei Colli - Ospedale Monaldi, Naples, Italy; 3 Department of Precision Medicine, University of Campania “Luigi Vanvitelli”, Naples, Italy; 4 Unit of Clinical Biochemistry, A.O.R.N. Ospedali dei Colli - Ospedale Monaldi, Naples, Italy

**Keywords:** Heart valve disease, Biomarkers, Mortality, Heart failure, Organ dysfunction

## Abstract

**Background:**

Infective endocarditis (IE) is a life-threatening disease whose prognosis is often difficult to predict based on clinical data. Biomarkers have been shown to favorably affect disease management in a number of cardiac disorders. Aims of this retrospective study were to assess the prognostic role of procalcitonin (PCT), pro-adrenomedullin (pro-ADM) and copeptin in IE and their relation with disease characteristics and the traditional biomarker C-reactive protein (CRP).

**Methods:**

We studied 196 patients with definite IE. Clinical, laboratory and echocardiography parameters were analyzed, with a focus on co-morbidities. PCT, pro-ADM and copeptin were measured on stored plasma samples obtained on admission during the acute phase of the disease.

**Results:**

Pro-ADM and copeptin were significantly higher in older patients and associated with prior chronic kidney disease. Pro-ADM was an independent predictor of hospital mortality (OR 3.29 [95%C.I. 1.04–11.5]; *p* = 0.042) whilst copeptin independently predicted 1-year mortality (OR 2.55 [95%C.I. 1.18–5.54]; *p* = 0.017). A high PCT value was strictly tied with *S. aureus* etiology (*p* = 0.001). CRP was the only biomarker associated with embolic events (*p* = 0.003).

**Conclusions:**

Different biomarkers correlate with distinct IE outcomes. Pro-ADM and copeptin may signal a worse prognosis of IE on admission to the hospital and could be used to identify patients who need more aggressive treatment. CRP remains a low-cost marker of embolic risk. A high PCT value should suggest *S. aureus* etiology.

**Supplementary Information:**

The online version contains supplementary material available at 10.1186/s12879-020-05655-7.

## Background

Infective endocarditis (IE) is a life-threatening disease with a mortality essentially unmodified over the last decades, despite of the improvement in medical and surgical care [1, 2]. The use of biomarkers able to predict the severity of this disease as well as the short- and mid-term prognosis could be very useful to tailor the therapeutic approach.

C-reactive protein (CRP) has long been the key marker of inflammatory, thrombotic and infectious diseases, although showing suboptimal specificity [3]. Recently, other molecules have been studied as biomarkers of bacterial infection and/or short term prognosis. Among them, procalcitonin (PCT), pro-adrenomedullin (pro-ADM) and copeptin seem to be the most promising based on existing evidence.

PCT, a calcitonin precursor synthetized by almost all human tissues, is considered a valid marker of the host response to acute bacterial infection, based on its synthesis and kinetics [4–6]. In addition, PCT often prompts initiation of antimicrobial therapy and guides antibiotic treatment de-escalation and/or discontinuation [7–9], also in critically ill patients [10]. PCT has been recently designated as novel biomarker for cardiologists, helping to discriminate the possible infectious origin of conditions such as dyspnea, congestion, valve disease, and acute coronary syndromes [11].

Pro-ADM is a stable precursor of ADM, a vasodilatator and natriuretic peptide, secreted by endothelial cells and vascular smooth muscle cells. It shows elevated levels in sepsis and is considered a marker of mortality in septic and nonseptic shock [12–15].

Copeptin, a precursor of pre-pro-vasopressin, is an established biomarker in different conditions of cardiovascular injury, including myocardial infarction, heart failure and stroke [16–19]. More recently, copeptin levels have been studied in sepsis and septic shock in children and adults [20, 21]. Higher copeptin levels on admission to the hospital were present in non survivors and, consequently, considered as a negative prognostic marker in adults with sepsis [21].

PCT, proADM and copeptin have different specificity and sensitivity in the course of infectious syndromes and, taken together, could improve the diagnostic and follow-up pathways of patients with IE. While the role of PCT in IE has been the subject of a few studies, and remains controversial [22–24], no data are available at present on the dynamics and possible utility of pro-ADM and copeptin in IE.

Accordingly, the aim of the present study was to assess whether PCT, proADM and copeptin have a prognostic value in IE and may predict IE outcome. Moreover, we studied the relation of these biomarkers with IE clinical features, microbial etiology, other inflammation/infection markers, i.e. CRP, as well as liver and kidney function.

## Methods

### Study design

This was a retrospective study conducted at the Unit of Infectious & Transplant Medicine, Monaldi Hospital, University of Campania “Luigi Vanvitelli”, involving patients with a diagnosis of IE admitted between 2007 and 2019 and for whom a plasma sample obtained in the acute phase of the disease was available. IE diagnosis was done according to existing criteria over time (modified Duke criteria until 2014 and ESC criteria from 2015 on) [25, 26]. All patients hospitalized at our Unit with diagnosis of IE undergo blood sampling. In the period 2007–2019 we observed in our Hospital, a regional referral center for IE, 525 IE cases. We included in this study all adult patients (*n* = 196) who were admitted to our Unit with a recent diagnosis of IE and without need of emergent surgery. They were referred from other Departments of our hospital (4 Cardiology units), other hospitals and after observation as outpatients. Most referred patients (*n* = 327) were excluded, although we had a sample available, because this sample was a later sample during the course of the disease. No patient with shock or septic shock was included, as these are routinely admitted to the Intensive Care Unit (ICU), not our Unit.

The study was approved by the Ethics Committee of the University of Campania “Luigi Vanvitelli” and AORN Ospedali dei Colli. All patients gave their written informed consent to blood sampling and the anonymous use of their clinical data.

### Patients included

Detailed data of patients were available as part of a standardized protocol of IE evaluation in use at our Unit, which includes a baseline clinical evaluation, particularly clinical history, physical examination, body mass index (BMI), chest X-ray, abdominal ultrasound scan and laboratory analyses (including CRP, creatinine, urea, glycemia, blood count, INR). According to the protocol, a trans-thoracic echocardiogram (TTE) was performed in all patients within 72 h of admission, followed by a trans-esophageal echocardiogram (TEE) where needed. Detailed information about IE characteristics (on native, prosthetic or cardiac implantable electronic device [CIED]), endocardial vegetations (number, size and position) and isolated causative pathogens were also collected. Embolic events, defined as acute complications causing overt clinical manifestations [27], and their characteristics (location, extension, complications) were also recorded.

Chronic heart failure (CHF), chronic kidney disease (CKD), liver disease and diabetes mellitus were considered as the principal co-morbidities. The Charlson comorbidity index (CCI) was also calculated for each patient.

PCT, pro-ADM and copeptin were measured in included patients on plasma samples, as detailed below. The obtained values of these biomarkers were analysed in relation with clinical (age, sex, embolism, involved valves, co-morbidities) and laboratory (CRP, white blood cells, creatinine, glycemia, alanine transferase (ALT), isolated microorganism) parameters. The estimated glomerular filtration rate (eGFR) was calculated by Modification of Diet in Renal Disease (MDRD) formula. In addition, PCT, pro-ADM and copeptin plasma levels were studied in relation to short term (at hospital discharge) and long term (at 1 year from the IE diagnosis) mortality.

### Biochemical assays

Blood was collected in EDTA tubes (Greiner Bio-One, Kremsmünster, Austria), cooled at 4 °C and centrifuged; plasma samples were stored at − 80 °C at the time of IE diagnosis, for subsequent use.

Plasma concentration of pro-ADM (cut-off for positivity 0.38 nmol/L), copeptin (cut-off for positivity 3.9 pmol/L) and PCT (cut-off for positivity 0.064 μg/L) were measured using an automated immunofluorescent assay on a Kryptor system (B.R.A.H.M.S. AG, Henningsdorf, Germany).

Other laboratory parameters were obtained by routine methods used in our Hospital central laboratory, including C-reactive protein (cut-off for positivity 0.3 mg/dL).

### Statistical analysis

Numerical data are presented as median with range, whilst categorical/nominal data as number and percentage. The Mann-Whitney U test was used to assess statistical significance of the differences between numerical groups of variables and the Fisher’s exact test for nominal variables. Logistic regression analysis of independent predictors of hospital mortality and 1-year mortality was performed by block entering in the model all variables significantly associated with each of these outcomes on the univariate analysis. Correlation between numerical variables was assessed by Spearman’s coefficient. The biomarkers pro-ADM, copeptin, PCT and CRP have been studied altogether for the first time in IE and no specific cut-off correlated to short and long term outcome of the disease was available. Using as positivity cut-offs the biomarker values indicated on the manufacturer instruction manual as reported in the methods section, most patients had positive values (pro ADM 185 vs 11, PCT 169 vs 27, copeptin 177 vs 19, CRP 187 vs 9). Accordingly, we evaluated each biomarker using as cut-off its median value observed in the overall study group.

To assess the predictive performance of biomarker levels on IE outcome, we calculated the area under the Receiver Operating Characteristic (ROC) curve, entering in-hospital mortality as the state variable. The significance level was set at 5% and all tests were 2-tailed. All analyses were performed using the statistical software for Windows SPSS 20 (SPSS, Inc., Chicago, Illinois, USA).

## Results

One-hundred and ninety-six patients were included. Median age was 62.3 years and 71% were males. General characteristics of patients are shown in Table 1.

**Table 1:** Baseline clinical features of the 196 IE patients studied

Number	196
Age, yrs., median [range]	62.3 [16–87]
Male gender, number (%)	139 (70.9)
Body Mass Index, kg/m^2^, median [range]	25.5 [15.4–39.1]
Chronic Heart Failure (prior to IE onset), number (%)	53 (27.0)
Diabetes mellitus, number (%)	34 (17.3)
Liver Disease, number (%)	23 (11.7)
Chronic Kidney Disease, number (%)	26 (13.3)
White blood cells, cells/μL, median [range]	9700 [3520–29,120]
Platelets, cells/μL, median [range]	209,000 [16000–812,000]
Hemoglobin, g/dL, median [range]	10.9 [6.6–17.7]
Creatinine, mg/dL, median [range]	1.0 [0.3–9.5]
Glucose, mg/dL, median [range]	105 [10–337]
Troponin I, ng/mL, median [range]	0.05 [0.0–92.5]
D-Dimer, ng/mL, median [range]	616 [5–16,640]
International Normalised Ratio, median [range]	1.0 [1.0–8.0]
Fibrinogen, mg/dL, median [range]	438.5 [103–1346]
Erythrocyte sedimentation rate, mm/h, median [range]	53 [2–124]
C-reactive protein, mg/dL, median [range]	6.1 [0.1–69.0]
Pro-Adrenomedullin, nmol/L, median [range]	1.05 [0.01–13.1]
Procalcitonin, μg/L, median [range]	0.16 [0.02–193.5]
Copeptin, pmol/L, median [range]	12.5 [2.14–389]
Vegetation location, number (%):	
*Aortic valve*	68 (34.7)
*Mitral valve*	47 (24.0)
*Tricuspid/Pulmonary valve*	15 (7.7)
*Cardiac Implantable Electronic Device*	40 (20.4)
*Multivalve involvement*	22 (11.2)
*Other*	4 (2.0)
IE subtype, number (%):	
*Native valve*	98 (50.0)
*Prosthetic valve*	47 (24.0)
*Repaired valve*	2 (1.0)
*Cardiac Implantable Electronic Device*	45 (23.0)
*Other*	4 (2.0)
IE Causative Pathogen, number (%)	
*Streptococci*	62 (31.6)
*Coagulase-negative Staphylococci*	38 (19.4)
*Staphylococcus aureus*	31 (15.8)
*Negative cultures*	28 (14.3)
*Enterococci*	28 (14.3)
*Other pathogens*	9 (4.6)
Positive blood culture bottles, number (%)	3.0 [0–12]
Vegetation size (long. dimension), mm, median [range]	14 [< 0.1–46.0]

IE occurred more frequently on the aortic valve and on native valves, and Staphylococci and Streptococci were the most frequent causative pathogens isolated. In-hospital mortality rate was 12.3%, 1-year mortality rate was 30% and there were 7 patients (3.5%) lost to follow-up.

Median levels of CRP, pro-ADM, PCT and copeptin are shown in Table 1. Strong correlations among the different biomarkers were observed (see Additional Table 1). The strongest linear correlation was observed between pro-ADM and copeptin (r = 0.629; *p* < 0.001). All four biomarkers correlated with D-dimer levels and, except CRP, with both NT-pro-BNP and creatinine, suggesting a relation with cardiac dysfunction and a strong influence of a declining renal function on biomarker levels. No significant difference was observed in copeptin levels according to gender (12.1 pg/ml in males vs 15.0 pg/ml in females; *p* = 0.96). None of the study patients had any sign of diabetes insipidus or the syndrome of inappropriate antidiuretic hormone.

### Biomarkers and IE mortality

Biomarker levels were evaluated in relation to major clinical features and hospital mortality of IE patients (Table 2). At univariate analysis, pro-ADM and copeptin were significantly higher in older patients. In-hospital mortality was significantly associated with higher levels of pro-ADM, PCT and copeptin, while 1 year mortality was significantly associated only with higher levels of pro-ADM and copeptin (Table 2).

**Table 2:** Biomarker levels in relation with major clinical features and mortality of IE patients §

	Pro-Adrenomedullin	^*p*-value	Procalcitonin	^*p*-value	Copeptin	^*p*-value	C-reactive protein	^*p*-value
*Gender*								
*Male*	0.98 [0.11–13.09]	0.431	0.16 [0.03–19.89]	0.841	12.1 [2.14–389]	0.966	6.0 [0.10–69]	0.505
*Female*	1.27 [0.01–7.79]		0.17 [0.02–193.5]		15 [2.29–157.10]		6.2 [0.10–46]	
*Age*								
*≤ 62.3*	0.75 [0.11–13.09]	**< 0.001**	0.145 [0.02–19.89]	0.345	8.98 [2.14–389]	**< 0.001**	6.4 [0.10–29.8]	0.866
*> 62.3*	1.32 [0.01–7.79]		0.185 [0.04–193.5]		21.6 [2.49–202.10]		5.9 [0.10–69]	
*Embolic event*								
*No*	1.09 [0.05–13.09]	0.733	0.15 [0.02–19.89]	0.083	12.9 [2.14–389]	0.878	5.2 [0.10–69]	**0.004**
*Yes*	0.98 [0.01–7.79]		0.20 [0.02–193.5]		12.0 [2.29–233.40]		7.70 [0.10–29.8]	
*Surgery Indication*								
*No*	0.98 [0.01–7.79]	0.386	0.15 [0.04–193.5]	0.867	12.0 [2.49–157]	0.395	7.0 [0.5–46]	0.491
*Yes*	1.1 [0.11–13.09]		0.16 [0.02–19.89]		12.6 [2.14–389]		6.0 [0.10–69]	
*Surgery Performed*								
*No*	0.91 [0.01–7.79]	0.075	0.14 [0.04–193.5]	0.578	10.7 [2.49–233.4]	0.082	6.2 [0.1–46]	0.692
*Yes*	1.14 [0.11–13.09]		0.17 [0.02–19.89]		13.3 [2.14–389]		6.10 [0.10–69]	
*Hospital Mortality*								
*No*	0.95 [0.01–13.09]	**< 0.001**	0.15 [0.02–17.55]	**0.006**	11.7 [2.14–389]	**< 0.001**	5.9 [0.10–69]	0.056
*Yes*	1.94 [0.53–7.79]		0.32 [0.04–193.5]		35.7 [3.96–147.30]		7.2.2 [1.6–26.6]	
*1-Year Mortality*								
*No*	0.86 [0.01–13.0]	**< 0.001**	0.16 [0.02–17.5]	0.248	11.2 [2.14–389]	**< 0.001**	6.0 [0.10–69]	0.250
*Yes*	1.45 [0.05–7.79]		0.17 [0.04–193.5]		23.7 [3.70–225]		6.3 [0.10–27]	

§ data are median [range]; ^ *p*-value was generated by Mann Whitney U-test; 151 Surg. Indication – 128 Surg. Performed

We subsequently performed a multivariate logistic regression analysis to identify variables independently associated to IE outcomes. As shown in Table 3, panel A, only pro-ADM was an independent predictor of in hospital mortality (OR 3.29 [95%C.I. 1.04–11.5]; *p* = 0.042) (Table 3). In contrast, higher copeptin levels were independently associated to 1 year mortality (OR 2.55 [95%C.I. 1.18–5.54]; *p* = 0.017), while pro-ADM showed a trend for an association with this outcome (OR 1.93 [95%C.I. 0.9–4.12]; *p* = 0.088) (Table 3, panel B).

**Table 3:** Logistic regression analysis of variables associated with hospital mortality (panel A) and 1-year mortality (panel B)

A								
	Hospital Mortality		Univariate analysis			Logistic regression		
	No	Yes	Odds Ratio	(95% C.I.)	^p-value	Odds Ratio	(95% C.I.)	p-value
Age								
> 62.3 years	83	15	1.76	(0.73–4.25)	0.276			
≤ 62.3 years	88	9						
Gender								
Male	122	17	1.02	(0.40–2.68)	1.000			
Female	49	7						
Causative Pathogen								
Staph. aureus	28	3	1.37	(0.38–4.90)	0.772			
Other pathogens	143	21						
Infection Type								
Prosthetic	82	16	2.17	(0.88–5.34)	0.126			
Native	89	8						
Embolic Event*								
Yes	66	10	1.13	(0.47–2.70)	0.825			
No	105	14						
Surgery Indication								
Yes	130	20	1.57	(0.51–4.87)	0.606			
No	41	4						
Pro-Adrenomedullin								
> 1.05 nmol/L	77	20	6.10	(2.00–18.6)	< **0.001**	3.29	(1.04–11.5)	**0.042**
≤ 1.05 nmol/L	94	4						
Procalcitonin								
> 0.16 μg/L	77	18	3.66	(1.38–9.67)	**0.008**	2.23	(0.79–6.25)	0.127
≤ 0.16 μg/L	94	6						
Copeptin								
> 12.5 pmol/L	78	19	4.53	(1.61–12.6)	**0.002**	2.24	(0.71–7.11)	0.168
≤ 12.5 pmol/L	93	5						
C-reactive protein								
> 6.10 mg/dL	81	15	1.83	(0.76–4.40)	0.196			
≤ 6.10 mg/dL	89	9						
B								
	1-Year Mortality		Univariate analysis			Logistic regression		
	No	Yes	Odds Ratio	(95% C.I.)	^p-value	Odds Ratio	(95% C.I.)	p-value
Age								
> 62.3 years	58	39	2.76	(1.43–5.32)	**0.003**	1.91	(0.94–3.84)	0.071
≤ 62.3 years	74	18						
Gender								
Male	97	38	1.38	(0.70–2.71)	0.382			
Female	35	19						
Causative Pathogen								
Staph. aureus	20	9	1.05	(0.45–2.47)	1.000			
Other pathogens	112	48						
Infection Type								
Prosthetic	61	35	1.85	(0.98–3.48)	0.059			
Native	71	22						
Embolic event*								
Yes	52	21	1.11	(0.58–2.11)	0.871			
No	80	36						
Surgery Indication								
Yes	102	44	1.01	(0.48–2.10)	1.000			
No	30	13						
Pro-Adrenomedullin								
> 1.05 nmol/L	54	40	3.39	(1.74–6.60)	< **0.001**	1.93	(0.90–4.12)	0.088
≤ 1.05 nmol/L	78	17						
Procalcitonin:								
> 0.16 μg/L	63	29	1.13	(0.60–2.11)	0.752			
≤ 0.16 μg/L	69	28						
Copeptin								
> 12.5 pmol/L	54	42	4.04	(2.04–8.01)	< **0.001**	2.55	(1.18–5.54)	**0.017**
≤ 12.5 pmol/L	78	15						
C-reactive protein								
> 6.10 mg/dL	67	28	1.08	(0.58–2.02)	0.874			
≤ 6.10 mg/dL	64	29						

^ *p*-value was generated by Fisher’s exact test

* Includes stroke

Patients were divided on the basis of median values of each analyzed biomarker

**Fig. 1:**
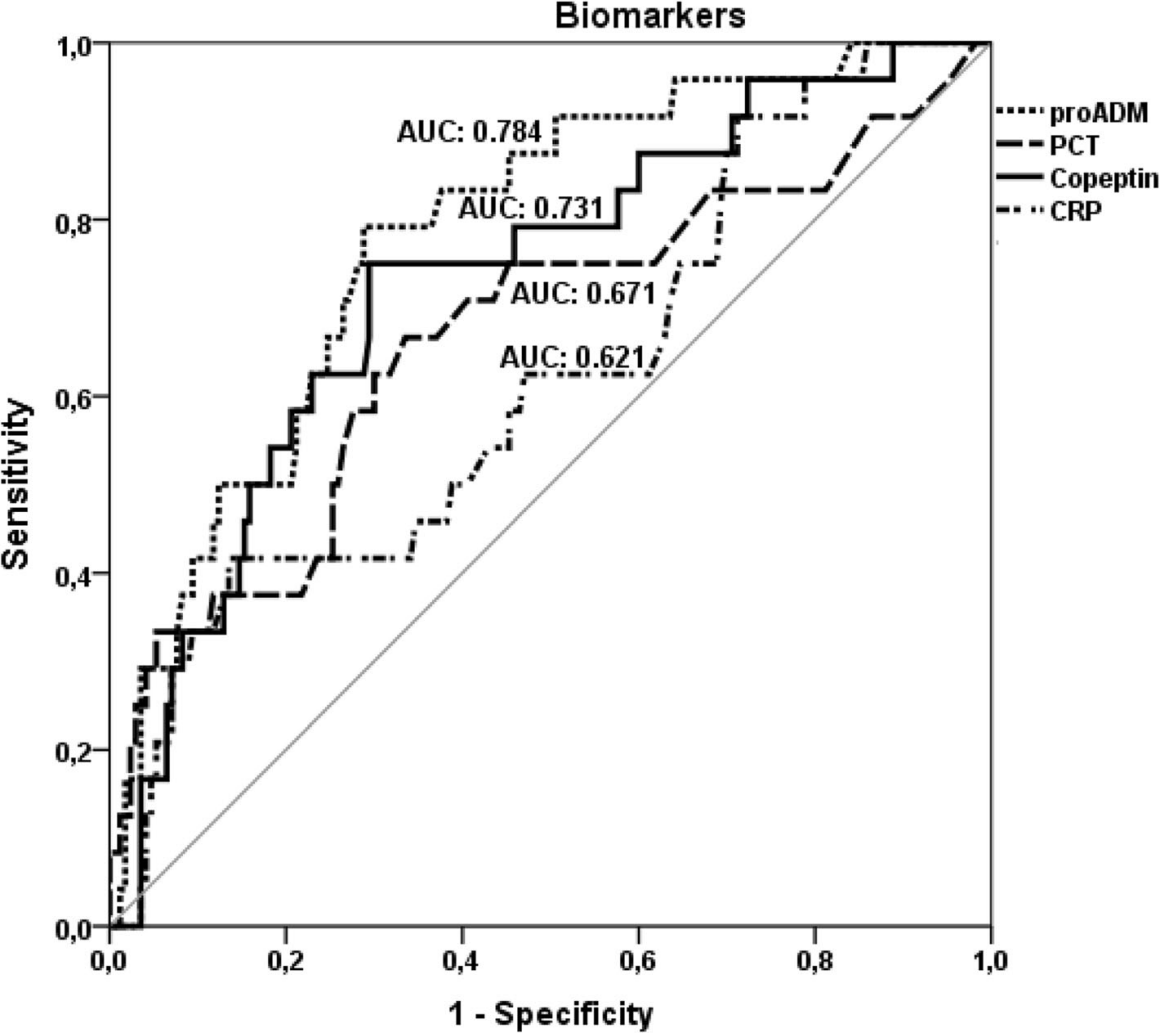
ROC curve analysis of the predictive value for in-hospital mortality of the four analysed biomarkers. Footnote: For each of the analysed biomarker the area under the Receiver Operating Characteristic (ROC) curve is shown. Predictive power for the designated outcome was highest for pro-adrenomedullin and lowest for C-reactive protein

At ROC curve analysis, pro-ADM and copeptin appeared to best predict in-hospital mortality (areas under the ROC curves: 0.784 and 0.731, respectively) compared with PCT and CRP (Fig. 1).

### Biomarkers and IE clinical features

Embolic events occurred in 77 patients (39.3%). CRP was the only biomarker associated with embolic events (*p* = 0.003) (Table 2). Most patients (151, 77%) had an indication for cardiac surgery at completion of diagnostic assessment, but this outcome was not related to any of the studied biomarkers (Table 2). Of these 151 patients, 128 (84.7%) finally underwent surgery, but actual surgical treatment was not associated with any biomarker level (Table 2).

Pro-ADM levels were higher in patients with left-sided IE compared to those with right-sided IE (*p* = 0.001; see Additional Table 2a), while no differences were observed for any biomarker in relation to the actual subtype of infection (native valve vs prosthetic valve vs CIED, see Additional Table 2b).

**Fig. 2:**
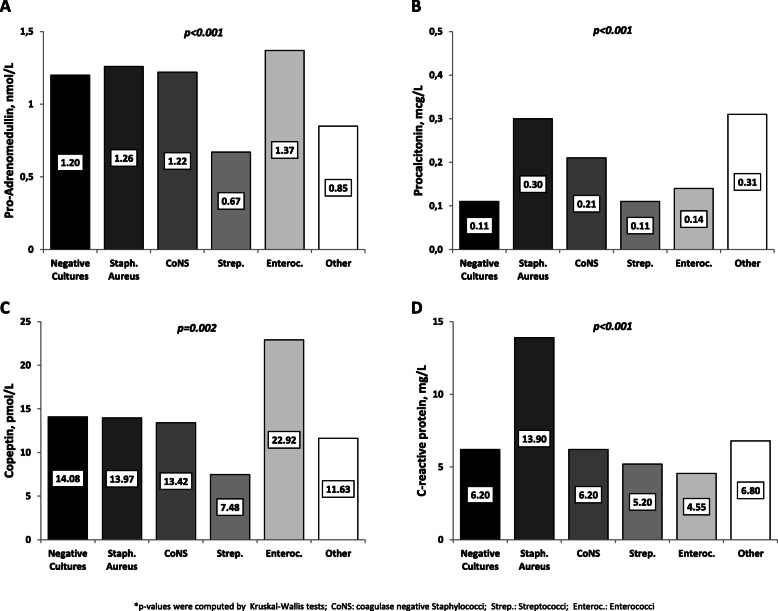
Biomarker levels according to IE causative pathogens. Footnote: Each bar depicts the median level of the designated biomarker among each subgroup of IE patients clustered according to the causative pathogen. The actual median value for plasma concentration is shown in the inset. Panel **a**: pro-adrenomedullin. Panel **b**: procalcitonin. Panel **c**: copeptin. Panel **d**: C-reactive protein

To evaluate association with IE etiology, we analyzed biomarker levels according to IE causative pathogen (Fig. 2). Interestingly, higher levels of pro-ADM were observed in patients with infection due to Enterococci and Staphylococci (Fig. 2a), whereas *Staphylococcus aureus* cases had the highest PCT levels (Fig. 2b). *S. aureus* IE cases also had higher CRP levels (Fig. 2d). The enterococcal etiology was associated to a significantly higher copeptin level (Fig. 2c). Streptococcal IE was characterized by the lowest biomarker levels (Figs. 2a-d2).

### Biomarkers and IE patient co-morbidities

Table 4 summarizes the relationship between biomarkers and co-morbidities. Liver disease and diabetes mellitus did not influence biomarker plasma concentration (Table 4). High levels of pro-ADM were significantly influenced by prior CHF and CKD while high levels of copeptin were associated with CKD only. CRP was also higher in the presence of CHF (*p* = 0.05). As shown in Additional Fig. 1, the CCI showed a direct correlation with pro-ADM and copeptin, but not CRP or PCT.

**Table 4:** Biomarkers and IE patient co-morbidities

	Pro-Adrenomedullin		Univariate analysis				Copeptin		Univariate analysis		
	***≤1.01 nmol/L >1.01***		***Odds Ratio***	***(95% C.I.)***	***^p-value***		***≤12.5 pmol/L >12.5***		***Odds Ratio***	***(95% C.I.)***	***^p-value***
*Heart Failure:*						*Heart Failure:*					
*No*	*81*	*62*	*2.76*	*(1.42-5.37)*	***0.004***	*No*	*77*	*66*	*1.77*	*(0.93-3.36)*	*0.107*
*Yes*	*17*	*36*				*Yes*	*21*	*32*			
*Chronic Kidney Disease:*						*Chronic Kidney Disease:*					
*No*	*94*	*76*	*6.80*	*(2.24-20.5)*	***<0.001***	*No*	*97*	*73*	*33.2*	*(4.39-250)*	***<0.001***
*Yes*	*4*	*22*				*Yes*	*1*	*25*			
*Diabetes Mellitus:*						*Diabetes Mellitus:*					
*No*	*85*	*77*	*1.78*	*(0.83-3.80)*	*0.186*	*No*	*85*	*77*	*1.78*	*(0.83-3.80)*	*0.186*
*Yes*	*13*	*21*				*Yes*	*13*	*21*			
*Liver Disease:*						*Liver Disease:*					
*No*	*88*	*85*	*1.34*	*(0.56-3.23)*	*0.658*	*No*	*86*	*87*	*1.10*	*(0.46-2.63)*	*1.000*
*Yes*	*10*	*13*				*Yes*	*12*	*11*			
*eGFR:*						*eGFR:*					
*>60mL/min*	*85*	*45*	*7.70*	*(3.80-15.6)*	***<0.001***	*>60mL/min*	*84*	*46*	*6.78*	*(3.39-13.5)*	***<0.001***
*≤60mL/min*	*13*	*53*				*≤60mL/min*	*14*	*52*			
	**Procalcitonin**		**Univariate analysis**				**C-reactive protein**		**Univariate analysis**		
	***≤0.16 μg/L >0.16***		***Odds Ratio***	***(95% C.I.)***	***^p-value***		***≤6.1 mg/dL >6.1***		***Odds Ratio***	***(95% C.I.)***	***^p-value***
*Heart Failure:*						*Heart Failure:*					
*No*	*70*	*73*	*1.46*	*(0.77-2.77)*	*0.262*	*No*	*65*	*77*	*1.95*	*(1.02-3.73)*	***0.050***
*Yes*	*31*	*22*				*Yes*	*33*	*20*			
*Chronic Kidney Disease:*						*Chronic Kidney Disease:*					
*No*	*92*	*78*	*2.22*	*(0.94-5.27)*	*0.091*	*No*	*82*	*87*	*1.69*	*(0.73-3.95)*	*0.292*
*Yes*	*9*	*17*				*Yes*	*16*	*10*			
*Diabetes:*						*Diabetes:*					
*No*	*85*	*77*	*1.24*	*(0.59-2.60)*	*0.578*	*No*	*84*	*77*	*1.55*	*(0.73-3.29)*	*0.263*
*Yes*	*16*	*18*				*Yes*	*14*	*20*			
*Liver Disease:*						*Liver Disease:*					
*No*	*90*	*83*	*1.18*	*(0.49-2.82)*	*0.825*	*No*	*87*	*85*	*1.11*	*(0.467-2.66)*	*0.828*
*Yes*	*11*	*12*				*Yes*	*11*	*12*			
*eGFR:*						*eGFR:*					
*>60mL/min*	*78*	*52*	*2.84*	*(1.51-5.19)*	***0.001***	*>60mL/min*	*63*	*66*	*1.11*	*(0.61-2.01)*	*0.763*
*≤60mL/min*	*23*	*43*				*≤60mL/min*	*34*	*32*			

^*p*-value was generated by Fisher’s exact test; patients were divided on the base of median values of each analyzed biomarker; *eGFR* estimated glomerular filtration rate

We also assessed whether an IE-related kidney dysfunction influenced biomarker levels. As shown in Table 4, all biomarkers were significantly higher in patients with a baseline eGFR < 60 ml/min, except CRP.

Comorbidities were also associated with clinical outcomes (Additional Table 3). In particular, kidney disease and diabetes were associated with hospital mortality, whereas heart failure and kidney disease were related with 1 year mortality. Also, the CCI was significantly associated with hospital mortality (median 4 [0–13] in deceased vs 3 [0–13] in survivors; *p* = 0.023) as well as 1 year mortality (median 5 [0–13] in deceased vs 3 [0–13] in survivors; *p* < 0.001).

## Discussion

Biomarkers able to rate severity and predict outcome of IE could be useful in the management of the disease, possibly allowing to tailor the therapeutic approach. In this study, we evaluated the significance of PCT, proADM and copeptin levels in IE and their relation with a traditional biomarker such as CRP. Furthermore, we analysed their prognostic value to predict IE outcomes. Our data suggest that in the setting of IE biomarkers have a substantially distinct profile and signal different conditions.

### Biomarkers and prognosis of IE

To the best of our knowledge, this is the first study evaluating pro-ADM and copeptin in IE. As previously observed in other infectious syndromes [10, 13, 18, 19], pro-ADM and copeptin were strong and independent predictors of hospital and 1 year mortality, respectively, in IE. Pro-ADM levels showed a nearly 80% sensitivity for hospital death. In contrast, PCT did not predict IE mortality.

### Role of PCT and CRP in IE

PCT and CRP were tied to other disease features, including a staphylococcal etiology for PCT and the occurrence of embolic events for CRP.

Our data fill the knowledge gap on the role of PCT in IE, where controversial findings emerged in previous studies. PCT previously appeared a useful biomarker for the diagnosis of IE, and also showed a correlation with specific bacterial etiology and disease prognosis [22–24]. However, other studies failed to demonstrate superiority of PCT over CRP in predicting IE [23]. In our study, PCT showed a pattern similar, but not overlapping with CRP, but none had an independent predictive role for IE mortality. Only higher CRP concentrations confirmed to be associated with embolic events [27], a serious complication in IE.

### Biomarkers and IE etiology

Our results on the relation between biomarkers and IE etiology deserve comment. Different IE etiology may be associated with diverse risk factors, disease course, treatment response and time of referral. It was therefore interesting to observe how copeptin and pro-ADM, strongly increased in older patients, were associated with enterococcal IE, that typically occurs with increasing incidence in the multi-morbid elderly [28]. In agreement with other studies [22–24], we found that PCT levels were highest in infections due to *S. aureus* or gram negative bacteria.

### Biomarkers and co-morbidities

As many patients with IE have co-morbidities, it was interesting to evaluate their effect on/relation with biomarker levels. Pro-ADM and copeptin were higher in IE patients with a history of CKD and CHF, confirming the association of these biomarkers with organ failure [10, 13, 18, 19]. Moreover, pro-ADM and copeptin correlated with both NT-pro-BNP and creatinine, suggesting a relation with acute, IE-associated cardiac dysfunction, and also a strong influence of renal dysfunction on biomarker levels.

As mentioned, older age translated into higher pro-ADM and copeptin, and this could influence their effect on mortality. However, these biomarkers predicted mortality (either hospital or 1 year) independent of age.

### Study limitations

This was a single center, retrospective study and determination of the biomarkers pro-ADM, PCT and copeptin was performed on stored samples at a single time-point. The study sample was relatively small, although homogeneously managed according to existing guidelines.

Previous conflicting data suggested a key role for PCT in the assessment of antimicrobial therapy effectiveness in infectious syndromes other than IE [7–9]. Unfortunately, we could not analyze the on treatment dynamics of PCT to predict therapeutic response in this study.

Finally, we were unable to confirm our findings in a prospective validation cohort.

## Conclusions

Pro-ADM and copeptin may signal a worse prognosis of IE on admission to the hospital and provide information different from usual inflammatory biomarkers, such as CRP and PCT. CRP remains a low-cost marker of embolic risk and a tool to monitor treatment response. A high PCT value should suggest *S. aureus* etiology. A higher pro-ADM and copeptin level may instead identify IE patients for whom a more aggressive therapeutic approach could be warranted.

## Supplementary Information

**Additional file 1: Table S1.** Correlation coefficients (Spearman’ Rho) for several biomarkers analysed in the 196 IE patients studied.

**Additional file 2: Table S2.** Pro-adrenomedullin, Procalcitonin, Copeptin and C-reactive protein values according to the heart side affected (A) and the subtype IE (B).

**Additional file 3: Figure S1.** Biomarker levels according to different Charlson Comorbidity Index categories (0–1; 2–3; ≥4).

**Additional file 4: Table S3.** Effect of comorbidities on Hospital Mortality (A) and 1-Year mortality (B).

## Data Availability

The datasets used and/or analysed during the current study are available from the corresponding author on reasonable request.

## Abbreviations

CIED: Cardiac implantable electronic device
CHF: Chronic heart failure
CRP: C-reactive protein
CKD: Chronic kidney disease
IE: Infective endocarditis
NT-pro-BNP: N-terminal prohormone of brain natriuretic peptide:
PCT: Procalcitonin
pro-ADM: Pro-adrenomedullin
ROC: Receiver Operating Characteristic
BMI: Body mass index
TTE: Trans-thoracic echocardiogram
TEE: Trans-esophageal echocardiogram
eGFR: Estimated glomerular filtration rate
MDRD: Modification of Diet in Renal Disease
ALT: Alanine aminotransferase
