# Supplementary material for: Prognostic value of pro-adrenomedullin and copeptin in acute infective endocarditis
Source: BMC Infect Dis. 2021 Jan 7;21:23. doi: 10.1186/s12879-020-05655-7 (PMC7791699; doi:10.1186/s12879-020-05655-7)
Supplement: Supplementary file 1 — Additional file 1: Table S1. Correlation coefficients (Spearman’ Rho) for several biomarkers analysed in the 196 IE patients studied. [file 12879_2020_5655_MOESM1_ESM.docx]

**Additional Table 1**

**Correlation coefficients (Spearman’ Rho) for several biomarkers analysed in the 196 IE patients studied**

|  | Pro-ADM | PCT | Copeptin | CRP | Platelet | Creatinine | Albumin | D-Dimer | NTproBNP | INR | Fibrinogen | CHO | BIL |
| --- | --- | --- | --- | --- | --- | --- | --- | --- | --- | --- | --- | --- | --- |
| Pro-ADM | 1.000 | 0.434^***^ | 0.629^***^ | 0.280^***^ | -0.227^**^ | 0.497^***^ | -0.415^***^ | 0.430^***^ | 0.643^***^ | 0.186^**^ | -0.019 | -0.253^*^ | 0.056 |
| PCT | 0.434^***^ | 1.000 | 0.286^***^ | 0.360^***^ | -0.268^***^ | 0.192^**^ | -0.329^**^ | 0.277^***^ | 0.246^**^ | 0.130 | -0.181 | -0.358^**^ | 0.195 |
| Copeptin | 0.629^***^ | 0.286^***^ | 1.000 | 0.080 | -0.172^*^ | 0.560^***^ | -0.445^***^ | 0.288^***^ | 0.456^***^ | 0.122 | -0.149 | -0.164 | 0.034 |
| CRP | 0.280^***^ | 0.360^***^ | 0.080 | 1.000 | -0.033 | -0.000 | -0.415^***^ | 0.324^***^ | 0.174 | 0.145^*^ | 0.387^***^ | -0.192 | 0.081 |

Pro-ADM=Pro-Adrenomedullin; PCT=Procalcitonin; CRP= C-reactive protein; NTproBNP= N-terminal pro-brain natriuretic peptide; INR= International normalized ratio; CHO= Total cholesterol; BIL= Total bilirubin.

*** Correlation is significant with a p <0.001

** Correlation is significant with a p <0.01

* Correlation is significant with a p<0.05
