# Supplementary material for: Prognostic value of pro-adrenomedullin and copeptin in acute infective endocarditis
Source: BMC Infect Dis. 2021 Jan 7;21:23. doi: 10.1186/s12879-020-05655-7 (PMC7791699; doi:10.1186/s12879-020-05655-7)
Supplement: Supplementary file 2 — Additional file 2: Table S2. Pro-adrenomedullin, Procalcitonin, Copeptin and C-reactive protein values according to the heart side affected (A) and the subtype IE (B). [file 12879_2020_5655_MOESM2_ESM.docx]

**Additional Table 2**

**Pro-adrenomedullin, Procalcitonin, Copeptin and C-reactive protein values according to the heart side affected (A) and the subtype IE (B)**

B

A

|  | *Left Sided IE*  *(n=117)* | *Right Sided IE*  *(n=55)* | *Multiple sites*  *(n=22)* | *^p-value* |
| --- | --- | --- | --- | --- |
| Pro-Adrenomedullin  >1.05 nmol/L, % | 52.1 | 28.1 | 19.0 | **0.001*** |
| Procalcitonin  >0.16 μg/L, % | 52.7 | 35.5 | 11.8 | 0.104 |
| Copeptin  >12.5 pmol/L, % | 58.3 | 27.1 | 14.0 | 0.391 |
| C-reactive protein  >6.1 mg/dL, % | 56.8 | 30.5 | 12.6 | 0.720 |

|  | *Native*  *(99)* | *Prosthetic*  *(47)* | *CIED*  *(45)* | *Other*  *(7)* | *^p-value* |
| --- | --- | --- | --- | --- | --- |
| Pro-adrenomedullin  >1.05 nmol/L, % | 48.0 | 25.5 | 23.5 | 3.1 | 0.945 |
| Procalcitonin  >0.16 μg/L, % | 49.5 | 18.9 | 27.4 | 4.2 | 0.230 |
| Copeptin  >12.5 pmol/L,% | 45.9 | 25.5 | 25.5 | 3.1 | 0.706 |
| C-Reactive Protein  >6.1 mg/dL, % | 52.6 | 24.7 | 20.6 | 2.1 | 0.683 |

*^p-value was generated by Pearson’s chi-squared test.*

*Significant differences were observed between group 1 and 2 and group 1 and 3 (Bonferroni method)

. *P-value was generated by Pearson chi-square test.*
