# Supplementary figures and images for: Prognostic value of pro-adrenomedullin and copeptin in acute infective endocarditis
Source: BMC Infect Dis. 2021 Jan 7;21:23. doi: 10.1186/s12879-020-05655-7 (PMC7791699; doi:10.1186/s12879-020-05655-7)

## Slide 1
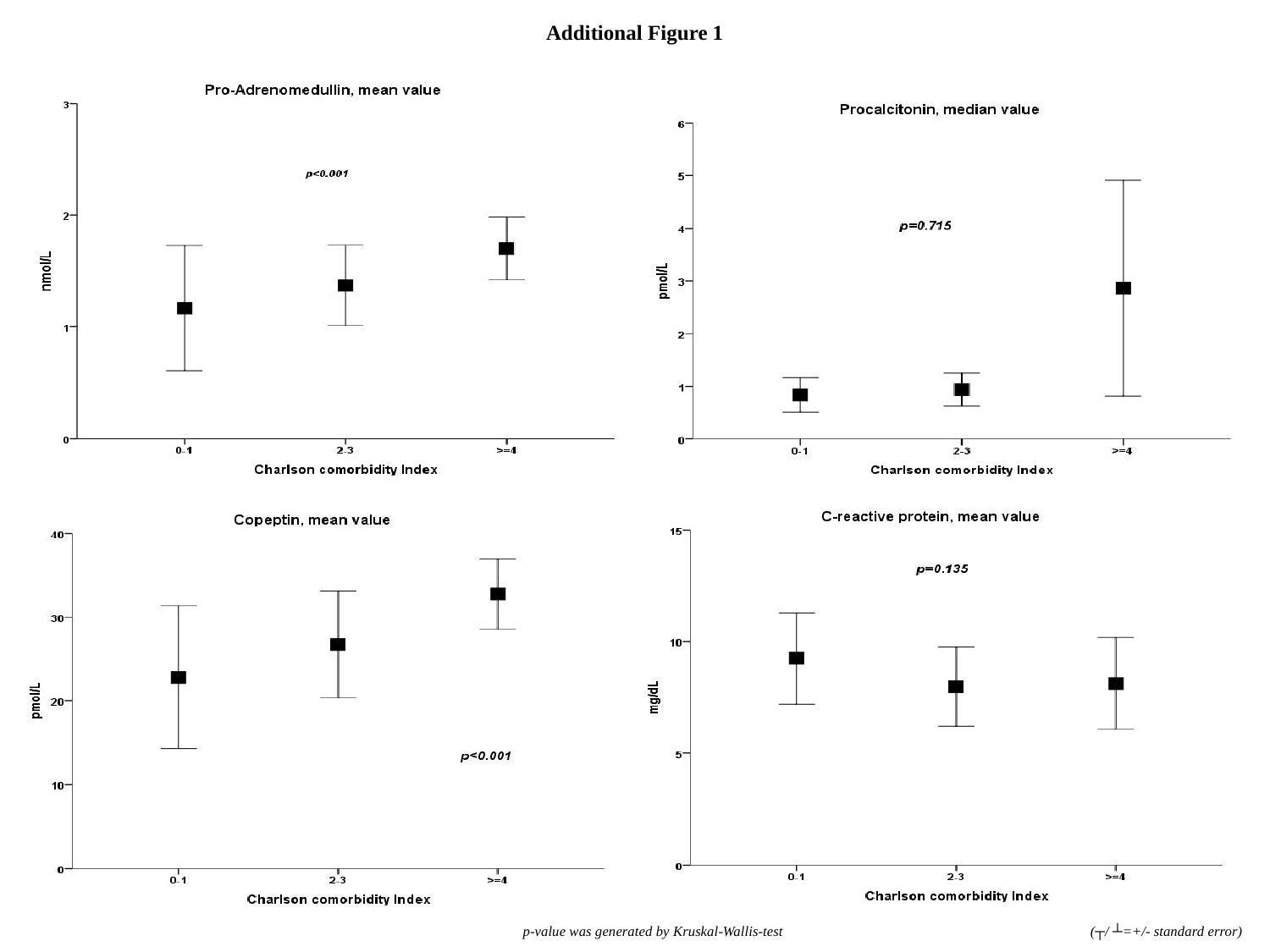

Additional Figure 1
p-value was generated by Kruskal-Wallis-test
(┬/ ┴=+/- standard error)

Supplement: Supplementary file 3 — Additional file 3: Figure S1. Biomarker levels according to different Charlson Comorbidity Index categories (0–1; 2–3; ≥4). [file 12879_2020_5655_MOESM3_ESM.pptx]
