# Supplementary material for: Prognostic value of pro-adrenomedullin and copeptin in acute infective endocarditis
Source: BMC Infect Dis. 2021 Jan 7;21:23. doi: 10.1186/s12879-020-05655-7 (PMC7791699; doi:10.1186/s12879-020-05655-7)
Supplement: Supplementary file 4 — Additional file 4: Table S3. Effect of comorbidities on Hospital Mortality (A) and 1-Year mortality (B). [file 12879_2020_5655_MOESM4_ESM.docx]

|  | Hospital Mortality  *No Yes* | | Univariate analysis  *Odds Ratio (95% C.I.) ^p-value* |
| --- | --- | --- | --- |
| *Heart Failure:*  *No*  *Yes* | *127*  *44* | *15*  *9* | *1.73 (0.70-4.23) 0.229* |
| *Chronic Kidney Disease:*  *No*  *Yes* | *154*  *17* | *15*  *9* | *5.43 (2.06-14.2)* ***0.001*** |
| *Diabetes Mellitus:*  *No*  *Yes* | *145*  *26* | *16*  *8* | *2.78 (1.08-7.18)* ***0.042*** |
| *Liver Disease:*  *No*  *Yes* | *153*  *18* | *19*  *5* | *2.23 (0.74-6.71) 0.171* |

**Additional Table 3**

**Effect of comorbidities on Hospital Mortality (A) and 1-Year mortality (B)**

A

|  | 1-Year Mortality  *No Yes* | | Univariate analysis  *Odds Ratio (95% C.I.) ^p-value* |
| --- | --- | --- | --- |
| *Heart Failure:*  *No*  *Yes* | *101*  *31* | *35*  *22* | *2.04 (1.05-3.99)* ***0.051*** |
| *Chronic Kidney Disease:*  *No*  *Yes* | *122*  *10* | *41*  *16* | *4.76 (2.00-11.3)* ***<0.001*** |
| *Diabetes Mellitus:*  *No*  *Yes* | *111*  *21* | *44*  *13* | *1.56 (0.72-3.38) 0.303* |
| *Liver Disease:*  *No*  *Yes* | *118*  *14* | *49*  *8* | *1.37 (0.54-3.48) 0.621* |

B

*^ p-value was generated by Fisher’s exact test*
